# Supplementary material for: Prevalence and predictors of anemia among six-week-old infants in Kwale County, Kenya: A cross-sectional study
Source: PLOS Glob Public Health. 2024 Mar 29;4(3):e0003062. doi: 10.1371/journal.pgph.0003062 (PMC10980238; doi:10.1371/journal.pgph.0003062)

PLOS Global Public Health

Prevalence and predictors of anemia among six-week-old infants in Kwale County, Kenya: A cross-sectional study

--Manuscript Draft--

Manuscript Number: PGPH-D-23-01767

Article Type: Research Article

Full Title: Prevalence and predictors of anemia among six-week-old infants in Kwale County, Kenya: A cross-sectional study

Short Title: Prevalence and predictors of anemia among six-week-old infants

Corresponding Author: Joyce Mwakishalua, Msc
Jomo Kenyatta University of Agriculture and Technology
Nairobi, KENYA

Order of Authors:
Joyce Mwakishalua, Msc
Simon Karanja
Raphael Lihana
Collins Okoyo
Nicole Stoffel
Michael Zimmermann

Keywords: Anemia; Infants; Iron deficiency; prevalence; risk factors; Kwale; Kenya

Abstract:

Background

Anemia is a significant public health problem among children worldwide. Risk factors for anemia are multifactorial but iron deficiency (ID) is the most common cause of anemia in low- and middle-income countries. ID and anemia in early infancy can impair growth and cognitive development. Therefore, detecting and correcting ID and anemia in early infancy is important. Many African infants have low birth weights and are born to iron deficient anemic mothers, and as a result, have low birth iron stores and are at risk of developing anemia early in infancy. The aim of this study was to determine the prevalence and predictors of anemia among six-week-old infants in Kwale County, Kenya.

Methods

This was a cross-sectional study among six-week-old infants including 424 mother-infant pairs. Structured questionnaires were administered to the mothers of the infants to obtain information on socio-demographic characteristics, maternal and birth information. Anthropometric data was collected for each child. A heel prick was done to measure hemoglobin and zinc protoporphyrin concentration using point-of-care devices. Prevalence of key nutritional conditions was done using binomial regression models. Chi-square was done to determine association between key variables. Univariable and multivariable regression analysis were done to determine factors associated with anemia. A spatial heat map was developed to show the spatial distribution of anemia cases in Kwale County.

Results

The overall prevalence of ID, anemia and IDA among infants in Kwale county was 60.4% (95%CI: 55.9-65.2), 21.0% (95%CI: 17.5-25.2) and 15.8% (95%CI: 12.7-19.7) respectively. Additionally, the prevalence of wasting, underweight and stunting were 2.1% (95%CI: 1.1-4.1), 0.2% (95%CI: 0.0-1.7) and 6.2% (95%CI: 4.3-9.0) respectively. Based on univariable analysis, male infants OR=2.20 (95%CI:1.33-3.63, p=0.002) and iron deficient infants OR=2.35, (95%CI: 1.39-3.99, p=0.001) were at significantly higher risk of anemia. Additionally, infants from Msambweni Sub- County OR=2.80 (95%CI: 1.70-4.62, p<0.001) were at higher risk for anemia compared to infants in Matuga Sub-

	County. Based on multivariable analysis, odds of anemia were significantly higher in infants born of mothers who did not use iron supplements during pregnancy, aOR=74.01 (95%CI: 2.45-2238.21, p=0.013) and odds of anemia were significantly lower in infants born of mothers with parity ≥ 4, aOR=0.05 (95%CI:0.00-0.77, p=0.024). Infants from Msambweni sub-County had significantly higher risk of anemia[[EQUATION]]([[EQUATION]] = 17.0, p<0.001) than infants from Matuga sub-County. Infant gender ([[EQUATION]] = 9.83, df=1, p=0.002), infant iron deficiency status ([[EQUATION]] = 10.46, df=1, p=0.001) and use of iron supplements during pregnancy ([[EQUATION]] = 4.48, df=1, p=0.034) were all significantly associated with infant anemia status.<br><br>Conclusion<br><br>In six-week-old infants in rural southern Kenya, anemia prevalence was 21.0% with ID accounting for 75.3% of anemia cases. Current Kenyan pediatric protocols specify provision of iron to anemic infants older than 6 months of age. Given the physical and cognitive impairments associated with ID and anemia in early infancy, it may be prudent to re-evaluate these pediatric protocols to include anemia screening and potential treatment of infants less than 6-months of age.
**Additional Information:**	
**Question**	**Response**
**Financial Disclosure**<br><br>Enter a financial disclosure statement that describes the sources of funding for the work included in this submission and the role the funder(s) played. This includes grants and any commercial funding of the work or authors.<br><br>This statement will be typeset if the manuscript is accepted for publication.<br><br>*Please review the submission guidelines and the instructions link below for detailed requirements and guidance.*	None
**Competing Interests**<br><br>On behalf of all authors, disclose any competing interests that could be perceived to bias this work.<br><br>This statement will be typeset if the manuscript is accepted for publication.<br><br>*Please review the instructions link below*	The authors involved in this study have no competing interest

and PLOS Global Public Health's competing interests policy to determine what information must be disclosed at submission.	
**Data Availability**<br><br>Before publication, Authors are required to make fully available and without restriction all data underlying their findings. Please see our PLOS Data Policy page for detailed information on this policy.<br><br>A **Data Availability Statement**, detailing where the data can be accessed, is required at first submission. Insert your Data Availability Statement in the box below.<br><br>Please see the data reporting section of our submission guidelines for instructions on what you need to include in your Data Availability Statement.<br><br>This statement will be typeset if the manuscript is accepted for publication.<br><br>*PLOS allows rare exemptions to address legal and ethical concerns. If you have legal or ethical restrictions, please use the box below to detail these in full sentences for the Journal team to consider.*	All data supporting the results and conclusions are provided within the manuscript.

Manuscript

Click here to access/download;Manuscript;Manuscript-Draft1.docx

Prevalence and predictors of anemia among six-week-old infants in Kwale County, Kenya: A cross-sectional study

Joyce Mwakishalua[1*], Simon Karanja[1], Raphael Lihana[2], Collins Okoyo[3,4], Nicole Stoffel[5], and Michael Zimmermann[6]

Affiliations

[1]School of Public Health, Jomo Kenyatta University of Agriculture and Technology, Nairobi, Kenya

[2]Centre for Virus Research (CVR), Kenya Medical Research Institute, Nairobi, Kenya

[3]Eastern and Southern Africa Centre of International Parasite Control (ESACIPAC), Kenya Medical Research Institute, Nairobi, Kenya

[4]Department of Epidemiology, Statistics and Informatics (DESI), Kenya Medical Research Institute, Nairobi, Kenya

[5]ETH Zurich, Laboratory of Human Nutrition, Institute of Food Nutrition and Health, Schmelzbergstrasse 7, CH-8092, Zurich, Switzerland.

[6]ETH Zurich, Laboratory of Human Nutrition, Institute of Food Nutrition and Health, Schmelzbergstrasse 7, CH-8092, Zurich, Switzerland.

*Corresponding author, email: stellawali507@gmail.com

Abstract

Background:

Anemia is a significant public health problem among children worldwide. Risk factors for anemia are multifactorial but iron deficiency (ID) is the most common cause of anemia in low- and middle-income countries. ID and anemia in [illegible] infancy can impair growth and cognitive development. Therefore, detecting and correcting ID and anemia in early infancy is important. Many African infants have low birth weights and are born to iron deficient anemic mothers, and as a result, have low birth iron stores and are at risk of developing anemia early in infancy. The aim of this study was to determine the prevalence and predictors of anemia among six-week-old infants in Kwale County, Kenya.

Methods:

This was a cross-sectional study among six-week-old infants including 424 mother-infant pairs. Structured questionnaires were administered to the mothers of the infants to obtain information on socio-demographic characteristics, maternal and birth information. Anthropometric data was collected for each child. A heel prick was done to measure hemoglobin and zin[illegible] protoporphyrin concentration using point-of-care devices. Preval[illegible] of key nutritional conditions was done using binomial regression models. Chi-square was done to determine association between key variables. Univariable and multivariable regression analysis were done to determine factors associated with anemia. A spatial heat map was developed to show the spatial distribution of anemia cases in Kwale County.

Results:

The overall prevalence of ID, anemia and IDA among infants in Kwale county was 60.4% (95%CI: 55.9-65.2), 21.0% (95%CI: 17.5-25.2) and 15.8% (95%CI: 12.7-19.7) respectively. Additionally, the prevalence of wasting, underweight and stunting were 2.1% (95%CI: 1.1-4.1), 0.2% (95%CI: 0.0-1.7) and 6.2% (95%CI: 4.3-9.0) respectively. Based on univariable analysis, male infants OR=2.20 (95%CI:1.33-3.63, p=0.002) and iron deficient infants OR=2.35, (95%CI: 1.39-3.99, p=0.001) were at significantly [illegible]er risk of anemia. Additionally, infants from Msambweni Sub- County OR=2.80 (95%CI: 1.70-4.62, p<0.001) were at higher risk for anemia compared to infants in Matuga Sub-County. Base[illegible] multivariable analysis, odds of anemia were significantly higher in infants born of mothers who did not use iron supplements during pregnancy, aOR=74.01 (95%CI: 2.45-2238.21, p=0.013) and odds of anemia were significantly lower in infants born of mothers with parity ≥ 4, aOR=0.05 (95%CI:0.00-0.77, p=0.024). Infants from Msambweni sub-County had significantly higher [illegible] of anemia ($\chi^2 = 17.0$, p<0.001) than infants from Matuga sub-County. Infant gender ($\chi^2 = 9.83$, df=1, p=0.002), infant iron deficiency status ($\chi^2 = 10.46$, df=1, p=0.001) and use of iron supplements during pregnancy ($\chi^2 = 4.48$, df=1, p=0.034) were all significantly associated with infant anemia status.

Conclusion:

In six-week-old infants in rural southern Kenya, anemia prevalence was 21[illegible] with ID accounting for 75.3% of anemia cases. Current Kenyan pediatric protocols specify provision of iron to anemic infants older than 6 months of age. Given the physical and cognitive impairments associated with ID and anemia in early infancy, it may be prudent to re-evaluate these pediatric protocols to include anemia screening and potential treatment of infants less than 6-months of age.

Key words: Anemia; Infants; Iron deficiency; Prevalence; Risk factors; Kwale; Kenya

Introduction

Anemia continues to be a significant public health problem in the world affecting over a third of the global popu[illegible]n with a worldwide prevalence of 22.8% across all ages (Gardner & Kassebaum, 2020) causing 68 million disability adjusted life years (DALYS) [2] . The World Health Organization (WHO) estimates that 42% of children < 5 years of age and 40% of pregnant women worldwide are anemic while one-third of all reproductive age women are anemic (WHO, 2022). R[illegible]nal estimates show that Sub-Saharan Africa bears the highest burden of anemia with a prevalence of 62% followed by South-East Asia (53%) while the Western pacific region has the lowest prevalence (21.9%) (WHO, 2022). Children under five years have the highest bu[illegible] of anemia, predominantly in low- and middle-income countries (LMIC) and more often it's due to ID as the mo[illegible]plicated cause accounting for ≥60% of the cases of anemia [2]. In Sub-Saharan Africa anemia is already common in infants < 1 year of age, as early as at the time of rou[illegible] vaccination; e.g., 70% of 6-month-old infants in south coastal Kenya are anemic [4].

Anemia of any etiology is associated with increased risks for child mortality and morbidity [5]. More specifically, iron deficiency anemia (IDA) in early infancy may result to irreversible cognitive development and cause physical retardation in growth [6]. Iron deficiency (ID) may also impair adaptive immunity and thereby reduce efficacy of vaccines in early infancy altho[illegible]he data on this is scanty and the immunology of malnutrition is not well understood [7][8]. As a result, IDA reduces the productivity of these infants as they grow through adolescence into adulthood since they have impaired cognitive ability and reduced physical development[9]. Other risk factors associated with anemia in the developing countries include: genetically acquired traits such as thalassemia and hemoglobinopathies, hemolysis or blood loss due to parasitic infections especially in malaria endemic areas, and other nutritional deficiencies including folate deficiency and vitamins A, C and B12 deficiencies[10]. Additionally, postnatal factors including frequent illnesses like pneumonia, earlier introduction of low iron content cereal-based complimentary foods, rapid postnatal growth rate, low birth weight as well as infectious diseases like malaria predispose infants to an early onset of ID state regardless of whether they were born at term or not [11].

In Kenya the nationwide prevalence of anemia in children under one year has been reported to be 39.5% [12]. Another study among infants 6 to 9 months in Keiyo sub-County found anemia prevalence to be 21.7% [13]. A study in Kwale County among infants aged 3-6months found the prevalence of ID, anemia and IDA to be 69%,52% and 38% respectively(Mikulic et al., 2020); but the study was small and non-representative.

Early infancy provides the window of opportunity for various health interventions such routine childhood immunization beyond which such interventions may not work and may result re-emergence of vaccine preventable diseases, poor growth as well as cognitive impairment with possible future health problems that may be irreversible [15][16]. In addition, ID may also be a previously unrecognized contributor to the reduced efficacy of vaccines in Sub-Saharan Africa [8]. Given these highlights, it is important to evaluate the role of anemia in early infancy as a contributor to high child mortality and morbidity in Sub-Saharan Africa.

Provision of iron supplements to infants and children 6-24 months of age has been recommended in areas where anemia is a severe public health problem i.e., prevalence ≥40 % (WHO, 2016). However, infants below six months of age have been exempted from many recommendations targeting prevention and treatment of ID and anemia in highly prevalent anemia regions. This is because it has generally been assumed that infants during their first six months of life are protected from IDA because they have accumulated adequate body iron stores during fetal life and therefore anemia is not a problem at this age [18]. However, this may not be the case in sub-Saharan Africa because sufficient iron is passed from the mother to the fetus only if the mother has adequate iron stores during pregnancy and most pregnant women in their third trimester in Africa are anemic and iron deficient [2]. Although delayed cord clamping has recently been proposed to prevent ID and the resulting anemia in infants, it has been difficult to implement in sub-Saharan Africa because of weak healthcare systems and busy maternity wards [19].

Despite all our knowledge on anemia and ID in children, researchers point out that there is much that remains unknown about this problem in early infancy. Previous studies on anemia in Kenya have been restricted to infants above six months of age (Ngesa & Mwambi, 2014;Onyangore et al., 2016;Jaeggi et al., 2013). Moreover, prior studies on anemia in malaria endemic regions in LMIC who have the highest burden of anemia in younger children have not included geographical factors in assessing the relationships between such outcomes and ecological as well as geographic factors [20]. The aim of this study therefore, was to determine

the prevalence of anemia and ID with associated predictors among six-week-old infants in Kwale County, Kenya with a global aim of improving infants' nutritional status in early life.

Materials and Methods

Study design and setting

This was a cross-sectional study conducted in Msambweni County Referral Hospital and Kwale Sub-County H[illegible]al in Kwale County. The study was nested within a randomized clinical trial (RCT)(ref). The primary objective of the RCT was to determine whether detecting and correcting ID and IDA using well absorbed iron improves efficacy of vaccines and to define the specific immune mechanisms underlying this effect.

Kwale County is located in south coast of Kenya at Coordinates: 4°10′S 39°27′E bordering the Republic of Tanzania to the South West, Indian Ocean to the East, Taita-Taveta County to the West, Kilifi County to the North and Mombasa County to the North East. It has an estimated population of 866,820 (KNBS, 2019).

Msambweni County Referral Hospital is a Level 5 government health facility. It offers both inpatient and outpatient medical services and has a bed capacity of 155. Being a referral hospital, it receives several cases from different areas within the county and therefore a good representative of the county population.

Kwale Sub-County Hospital is a high-volume level 4 government health facility within Matuga Sub-County. It has a bed capacity of 62 and offers both inpatient and outpatient medical services.

Sample size determination

Sample size was calculated using Fisher *et al* formula (1998); $n=(z^2pq)/e^2$; where Z is the value from standard normal distribution corresponding to 95% confidence level (Z=1.96), p is the estimated prevalence of anemia among infants in Kwale County (assumed as 50%), q is the proportion of infants without anemia (1-p), e is the margin of error assumed as 5%. The final sample size was thus calculated as 424 mother-infant pairs after factoring in a 10% non-response rate. Simple random sampling was used to select the final sample size from the total number of mother-infant pairs who were eligible to be enrolled in the study. The participants were randomly sampled from the number of mother-infant pairs that came for screening for the RCT.

Survey procedures

The study was carried out between January and April 2023. During recruitment, the study purpose was explained to the mothers and those who were willing to participate were scheduled

for a screening date at the hospital when their child would be exactly 6 weeks old ± 3 days. On the day of screening, the study aim was explained again in detail to the mot[illegible] who signed consent forms both in English and Swahili and each mother-infant pair given a unique identification number. A screening form was programmed onto redcap and administered using tablets. The study included HIV negative mothers above 15 years of age with infant aged 6 weeks ± 3 days, born via vaginal delivery, full-time breastfed, not having received any vaccine beyond the birth dose of Oral Polio Vaccine (OPV) and Bacillus Calmette-Guerin (BCG), and with no medical condition that precludes study involvement. The study excluded infants with conditions that might have affected participation such as hematological and non-hematological malignancies, chronic kidney disease, diabetes and inability to provide maternal informed consent. Mother-infant pairs who met the inclusion criteria were enrolled into the study.

Data collection

An interviewer administered questionnaire was used to collect data on socio demographic anc[illegible] maternal characteristics. Date of birth, maternal age, hemoglobin (Hb) level during last ANC visit and parity and other factors were subsequently confirmed from the mother-child ANC booklet or immunization card or exercise book. Standardized flat Seca 813 digital measurement scale by German SECA manufacturers was used to measure infant's body weight to the nearest 0.01kg. Standard measurement board by UNICEF was used to measure infants' length/height to the nearest 0.5cm. A heel prick was done using single use sterile lancet; then Hb concentration measured on capillary blood using Hemocue 301 system device to determine anemia status at an absorbance between 565 and 880nm and the concentration expressed as grams per/deciliter(g/dl). ZnPP concentration was measured on capillary blood using iCheck Anemia device (Bioanalyt, Germany) to determine ID status. The device uses photometric determination of ZnPP by auto florescence. The measured ZnPP value is a ratio expressed as µmol/mol Hb; (ZnPP value displayed on the machine in milligrams per litre (mg/l) was divided by Hb level (g/dl) and then multiplied by a constant factor 2574). Both machines have quality control solutions (two levels; High and Low) which were performed prior to measuring samples during screening in compliance to point-of-care testing guidelines.

Data management and analysis

The WHO anthropometrics software was us[illegible] calculate infants Z-scores for weight for length (WLZ)-wasting, weight for age (WAZ)-underweight and length for age (LAZ)-stunting. Both questionnaires and laboratory diary information were programmed into tablets then used

to capture data electronically on Redcap which had inbuilt data quality checks to reduce errors during the data entry process. Collected data was sent to the server daily. At [illegible]nd of the data collection period, all data was downloaded onto MS Excel then transported to Stata version 16 for analysis. Descriptive analysis was done to describe characteristics of the participants. Summary tables were used to show proportion of infants with selected nutritional conditions such as wasted, underweight, stunted, ID, anemic and those with IDA by different factors. Chi square test was used to show association between predictor variables and anemia[illegible]us. Both maternal and infant factors related to anemia were analyzed on univariable analysis and strength of association reported a[illegible]ds ratio (OR) with 95% confidence intervals (CI). Variable multicollinearity was done to identify variables that were strongly correlated. To select variables for multivariable analysis an inclusion criterion of p-value <0.5 was used in a stepwise variable selection method. A multivariable [illegible]ysis was done using the selected variables and strength of association reported as adjusted OR (aOR) with 95% CI. Sub-County, ward (location) and village of residence of the infants in the study was be displayed on spatial heat map to show the spatial distribution of anemia cases in Kwale County.

Ethical approval and consent to participate

Approval for the study was sought from the JKUAT Institutional Ethics review Committee (approval number; JKU/IERC/02316/0052). Additional approval was provide[illegible] the Kwale county health authorities after they were appropriately briefed about the study. At the facility level written participant consent was obtained from the mothers.

Results

Sociode[illegible]raphic characteristics of participants

Overall [illegible],424 mother-infant pairs were enrolled in the study; 211 from Matuga sub-County and 213 from Msambweni sub-County. Of the 211 participants from Matuga sub-County, 82 (19.3%) were from Mkongani location, 117(27.6%) from Tsimba Golini location and 12 (2.8%) from Waa location. Of the 213 participants from Msambweni sub-County, 13 (3.1%) were from Kinondo location, 74 (17.5%) from Ramisi location, 100 (23.6%) from Ukunda location, and 26 (6.1%) from Gombato location (Table 1).

All infants in the study were aged 6 weeks (± 3 days) old. 55% of the infants were [illegible]e while 45% were female. The mean birth weight of the infants was 3.10 kg (SD=0.5kgs) ranging from 2.0 kg to 5.0 kg. Mean weight of the infants at the six weeks was 4.7 kg (SD=0.6kgs) and mean height was 54.7 cm (SD=2.1cm). Mean head circumference was 37.9 cm (SD=1.6cm) and average gestation weeks at birth was 37 weeks (SD= 1 week). Mean Hb

concentration of the infants was 12.3g/dl (SD=1.5g/dl). Mean ZnPP concentration of the infants was 44.0µmol/mol Hb (SD=13.1µmol/mol Hb).

Mean age of mothers in the study was 27.7 years (SD=6.1 years). Mean weight of the mothers was 59.8 kg (SD= 12.3 kg). The median number of children per mother was 3 with a minimum of 1 child and a maximum of 11 children. Mean Hb concentration of the mothers during the last ANC visit was 10.8 g/dl (SD=1.4 g/dl). Maternal information on education level and use of iron supplements during pregnancy was available for 157 mothers of whom 98.1% reported to having used iron supplements during pregnancy while 2% reported to not have used iron supplements during pregnancy. Mothers who had attained post-secondary level of education were 17.8%, those with secondary education level were 21.7%, those with primary education level were 54.1% and those with no education level were 6.4%.

Proportion of infants with selected nutritional conditions

The overall prevalence of ID, anemia and IDA among infants in Kwale county was 60.4% (95[illegible]I: 55.9-65.2), 21.0% (95%CI: 17.5-25.2) and 15.8% (95%CI: 12.7-19.7) respectively (Ta. Additionally, the prevalence of wasting, underweight and stunting were 2.1% (95%CI: 1.1-4.1), 0.2% (95%CI: 0.0-1.7) and 6.2% (95%CI: 4.3-9.0) respectively (Table 2).

Msambweni Sub-County had a higher prevalence of anemia 29.1% (95%CI:23.6-35.9) compared to Matuga Sub-County 12.8% (95%CI:9-18.2). Kinondo location in Msambweni Sub-County had the highest proportion of infants with anemia 50.4% (95%CI: 13.6-69.5) followed by Gombato Location 42.3% (95%CI:27.0-66.3) while Waa Location in Matuga Sub-County had no cases of anemia (Figure 1).

Male infants had a significantly higher prevalence of anemia 26.7% (95%CI:21.6-33.1) compared female infants 14.2% (95%CI:10.0-20.2). Additionally, infants with low birthweight had significantly higher prevalence of anemia 29% (95%CI:16.7-50.3) compared to infants with ≥ 2.5 kg birthweight, anemia prevalence 20.2% (95%CI:16.5-24.6). At six weeks of age infants who weighed < 5.0 kg had lower anemia prevalence 19.9% (95%CI: 15.8-25) compared to infants who weighed ≥5.0 kg, anemia prevalence 23.8% (95%CI:17.4-32.5). Similarly, infants whose head circumference was <38.0 cm had lower anemia prevalence 20.3% (95%CI: 16.0-25.7) compared to infants who had head circumference ≥38.0 cm, anemia prevalence 22.4% (95%CI:16.6-30.1). Based on gestational age at birth,

infants born <37 weeks gestation had higher anemia prevalence 24.5% (95%CI:15.3-39.3) compared to infants born ≥ 37 weeks gestation, anemia prevalence 20.5% (95%CI:16.7-25.1). Based on infants' height, infants whose height was <55.0 cm had higher anemia prevalence 22.0% (95%CI: 16.9-28.6) compared to infants with height ≥55.0 kg, prevalence 20.2% (95%CI:15.5-26.2).

Based on mothers age, the prevalence of anemia in infants 24.4% (95%CI:17.8-33.4) was significantly higher for mothers aged 31-40 years compared to anemia prevalence of 8.3% (95%CI: 1.3-54.4) for the category of mothers aged >40 years. By education level, mothers with primary education level had infants with significantly higher prevalence of anemia 22.4% (95%CI: 15.0-33.2) compared to mothers with no formal education [10.0% (95%CI: 1.6-64.2)]. Infants born of mothers of parity one had higher anemia prevalence 23.7% (95%CI: 16.4-34.1) compared to infants born of mothers of parity two [18.8% (95%CI: 12.8-27.6)]. With regard to maternal anemia status, mothers who were anemic during their last ANC visit had slightly higher proportion of infants with anemia 22.0% (95%CI: 16.6-29.1) compared to mothers who were not anemic during the last ANC visit 21.7% (95%CI: 16.2-19.1). Mothers weighing 50-59 kgs had a higher proportion of infants with anemia [26.0% (95%CI: 19.8-34.2)] compared to infants of mothers weighing >80 kgs [5.9% (95% CI 1.5-22.6)]. Infants born of mothers who reported to have used iron supplements during pregnancy had anemia prevalence of 18.2% (95%CI:13.0-25.4) compared to infants born of mothers who reported to have not used iron supplements during pregnancy 66.7% (95%CI:30.0-148.8).

Chi square analysis of factors associated with anaemia

Infants from Msambweni Sub-County had a significantly higher prevalence of anemia (χ^2 = 17.0, df=1, p<0.001) compared to infants from Matuga Sub-County.

Infants gender was significantly associated with anemia status (χ^2 = 9.83, df=1, p=0.002).

Infants iron deficiency status was significantly associated with anemia status (χ^2 = 10.46, df=1, p=0.001).

Use of iron supplements during pregnancy was significantly associated with infants' anemia status(χ^2 = [illegible], df=1, p=0.034) (Table 3).

Univariable analysis of factors associated with anaemia

Overall, infants in Msambweni Sub-County were 2.8 times, [OR=2.80 (95%CI: 1.70-4.62, p<0.001)] more likely to be anemic compared to infants in Matuga Sub-County.

Male infants had significantly higher odds of anemia compared to female infants, OR=2.20 (95%CI:1.33-3.63, p=0.002). Similarly, iron deficient infants also had significantly higher odds of anemia than iron replete infants, OR=2.35, (95%CI: 1.39-3.99, p=0.001). Mothers who did not use iron supplements during pregnancy had marginally non-significant higher odds of having infants with anemia than mothers who used iron supplements during pregnancy, OR=9.0 (95%CI:0.79-102.8, p=0.077) (Table 4).

Multivariable analysis of factors associated with anaemia

Pairwise correlation of variables in the univariable analysis was used to assess for correlation (Table 5). Stepwise variable selection with an inclusion criterion of p-value<0.5 was then done which included variables such as parity, birthweight, gender, age of the mother, use of iron supplements, education level and iron deficiency in the multivariable analysis model. Significantly higher odds of anemia were seen infants born of mot[illegible] who did not use iron supplements during pregnancy, aOR=74.01 (95%CI: 2.45-2238.21, p=0.013). Mothers with parity ≥ 4 had significantly lower odds of having infants with anemia aOR=0.05 (95%CI:0.00-0.77, p=0.024) (Table 6).

Discussion

This study examined 424 mother-infant pairs of six-week-old infants and found the prevalence of anemia, ID and IDA to be 21.0%, 60.4% and 15.8% respectively. Significant factors associated with anemia on univariable analysis were infants iron deficiency status, gender, mothers use of iron supplements during pregnancy and the sub-County of residence. On multivariable analysis, factors that remained significant were only two; use of iron supplements during pregnancy and parity of ≥4; possibly because of low anemia prevalence.

Anemia prevalence in this study is slightly lower than the global anemia prevalence of 22.8% across all ages (Gardner & Kassebaum, 2020) and 42% in children < 5 years of age [3]. Similarly, anemia prevalence in this study is lower than the prevalence in older Kenyan infants where 70% of infants at 6-months of age are anemic [4] and the nationwide anemia prevalence in Kenyan children under one year of 39.5% [12]. In additi[illegible]his prevalence is also lower compared to a prevalence of 69% in a recent non-representative study in the same area of Kenya among infants aged 6-10 months (Nyilima et al 2021). This is not surprising as younger infants are generally have high Hb levels in the first few weeks after birth due to high levels of circulating fetal hemoglobin (HbF); these levels decrease progressively over the first 6-mon[illegible] as HbF is replaced by adult hemoglobin (HbA) [23]. According to WHO categorization of

anemia the 21.0% anemia prevalence is of moderate public health significance and the[illegible]e it is important to reduce this burden to be of mild or of no public health significance (World Health Organization, 2016).

Prevalence of ID and IDA in this study was 60.4% and 15.8% respectively. This means ID accounted for 75.3% of the total anemia cases in this study. This agrees with documented global anemia burden where ID accounted for ≥60% of the cases of anemia in children <5 years in LMIC who predominantly have the highest burden of anemia [2]. A previous study among infants aged 3-6months in Kwale County found the prevalence of ID and IDA to be 69% and 38% respectively (Mikulic e[illegible] 2020); but the study was small and non-representative. IDA in early infancy may result to irreversible cognitive development and cause physical retardation in growth [6]. In addition, ID not only causes anemia but may also impair adaptive immunity and thereby reduce efficacy of vaccines in early infancy although the data on this is scanty and the immunology of malnutrition is not well understood [7][8].

Infant gender significantly predicted anemia status with male infants having an increased risk of anemia compared to female infants. Few studies have been done to address the question of gender differences on anemia and iron status in early infancy. Some studies have found higher anemia prevalence in boys compared to girls (61.3%) [24] [4]. These differences reflect increased cases of true ID state in boys while others point to genetics [25]. Anemia in infancy is associated with alterations in systemic metabolism dependent on gender with male infants having higher prevalence of anemia and ID due to greater oxidative stress and microbial dysfunction compared to female infants [26].

Iron supplementation in this study significantly predicted infants' anemia status with infants born of mothers who reported to have not used iron supplements during pregnancy having an increased risk of anemia compared to infants born of mothers who reported to have used iron supplements during pregnancy. This is in agreement with other clinical trials studies on effect of daily iron supplementation in conjunction with folic acid and other vitamins and minerals for pregnant women as an intervention in antenatal care, which showed that supplementation with iron reduced the risk of ID and maternal anemia [27]. During pregnancy, iron supplementation produces more benefits to both the mother and the baby by promoting fetal health, preparing the fetus with adequate iron stores to be used during early postnatal stages of life as well as reducing maternal morbidity (Georgieff et al., 2019).

In this study infants in Msambweni Sub-County had significantly higher risk of anemia compared to infants in Matuga Sub-County. While there was no direct measure of diet or other factors leading to this, this may be because of disparities in socio-economic status, health-seeking behaviors, access to health and nutrition literacy within Kwale County.

In this study infants of mothers with parity of four or more were significantly less likely to be anemic compared to infants born of mothers of parity one. This is in contrast to other studies where maternal parity has also been shown to be associated with childhood anemia with prevalence increasing as maternal parity increases [24]. However, these studies examined older infants from ages 6 months and older whereby many children in the family impacts the ability to feed them appropriately. The contrast in our study could be because mothers who have more than one child are more likely to comply and adhere to the importance of using iron supplements given during pregnancy through education and sensitization in previous pregnancies from health experts.

Whilst our study did not show any significant association between gestational age, birth weight and maternal anemia with infants' anemia status [illegible]her studies have shown significant associations. Preterm and low birthweight infants at a higher risk of developing ID and IDA because of higher iron requirements compared to term and normal birth weight infants due to increased post-natal growth rate [29]. A study in Bangladesh f[illegible]d significant association between maternal anemia and occurrence of childhood anemia with anemia prevalence among children born of anemic mothers being higher (62%) compared to children born of non-anemic mothers [30].

Study strengths and limitations

Major strength of this study was that it was population based as infants were recruited from villages surrounding the hospitals, maternity wards, well-baby clinics and vaccination clinics and therefore is likely more representative of the study area population. Hemocue 301 system device used in this study to determine anemia status is highly reliable and used routinely [illegible] clinical practice. The iCheck Anemia device (Bioanalyt, Germany) used in this study to determine ID status has been shown to be useful in screening for ID in field surveys especially in children because its measurement can be done directly with a single drop of

blood considering that infants in this study were only six-weeks old. In addition to infant related information maternal data was also collected to help determine their association with infants' anemia status.

Nevertheless, this study had several limitations including that it was nested within a parent study and therefore it included only infants who met the inclusion criteria for that study. Maternal information relied heavily on availability of mother-child health booklets which was not available for some mothers and also on the ability to remember and honestly disclose whether they used iron supplements during pregnancy or not. Participants were not recruited from household level but were mobilized to a central recruitment point within the county (i.e., hospitals, well-baby clinics and maternity wards).

Conclusion

Our study findings [illegible]gest that in rural Kenya, anemia in early infancy is of moderate public health significance affecting 21% of infants with ID accounting for 75.3% of these anemia cases. In addition, this study identified use of iron supplements as an important factor for reducing risk for infants' anemia. There is a need to address the problem of iron deficiency in early childhood to improve early infancy Hb levels and in the long-term avoid physical and cognitive impairments associated with iron deficiency in infancy. It would be prudent to re-evaluate current recommendations targeting provision of iron supplements to infants and children 6-24 months of age where anemia is a severe public health problem (prevalence ≥40%) to potentially include much younger infants given the high burden of ID in early infancy.

Declarations

Ethics approval

The approval for the study was sought from the Swiss Federal Institute of Technology (ETH) and the JKUAT Institutional Ethics review Committee, approval number; JKU/IERC/02316/0052. Additional approval was provided by the Kwale County health authorities. Written participant consent was obtained from the mothers.

Conflicts of interest

None of the authors involved in this study have a conflict of interest.

Competing interests

The authors involved in this study have no competing interest.

Availability of data and material

Data will be made available on request.

Authors contributions

Simon Karanja and Raphael Lihana were involved in conceptualization of the work, developing suitable methodology and provided supervision on data analysis. Collins Okoyo helped on data cleaning, analysis and data presentation. Nicole Stoffel and Michael Zimmermann were involved in drafting, editing and reviewing of the manuscript.

Acknowledgements

The authors are very grateful to the study participants and the entire Kwale community at large. Much appreciation to the County government of Kwale, especially the Minister of Health, nurses and clinicians for their support and co-operation during the study. Special thanks to the entire research team at Egemeo research limited led by Suzanne Nyilima for their tireless efforts in the recruitment of study participants and commitment towards this work.

References

1. Gardner W, Kassebaum N. Global, Regional, and National Prevalence of Anemia and Its Causes in 204 Countries. : 1990–2019. Available: https://academic.oup.com/cdn/article/4/Supplement_2/830/5845051
2. Kassebaum NJ, Jasrasaria R, Naghavi M, Wulf SK, Johns N, Lozano R, et al. A systematic analysis of global anemia burden from 1990 to 2010. Blood. 2014. doi:10.1182/blood-2013-06-508325
3. World Health Organization. No Title. 2022 [cited 24 Jan 2022]. Available: https://www.who.int/health-topics/anaemia#tab=tab_1
4. Jaeggi T, Moretti D, Kvalsvig J, Holding PA, Tjalsma H, Kortman GAM, et al. Iron Status and Systemic Inflammation, but Not Gut Inflammation, Strongly Predict Gender-Specific Concentrations of Serum Hepcidin in Infants in Rural Kenya. PLoS One. 2013;8. doi:10.1371/journal.pone.0057513
5. Brabin BJ, Premji Z, Verhoeff F. An analysis of anemia and child mortality. Journal of Nutrition. American Institute of Nutrition; 2001. doi:10.1093/jn/131.2.636s
6. Lozoff B, Beard J, Connor J, Felt B, Georgieff M, Schallert T. Long-lasting neural and behavioral effects of iron deficiency in infancy. Nutrition Reviews. 2006. doi:10.1301/nr.2006.may.S34-S43
7. Savy M, Edmond K, Fine PEM, Hall A, Hennig BJ, Moore SE, et al. Landscape analysis of interactions between nutrition and vaccine responses in children. Journal of Nutrition. 2009;139. doi:10.3945/jn.109.105312

8. Prendergast AJ. Malnutrition and vaccination in developing countries. Philosophical Transactions of the Royal Society B: Biological Sciences. 2015;370: 20140141. doi:10.1098/RSTB.2014.0141
9. Haas JD, Brownlie IV T. Iron deficiency and reduced work capacity: A critical review of the research to determine a causal relationship. Journal of Nutrition. 2001;131. doi:10.1093/jn/131.2.676s
10. Anaemia. [cited 31 Mar 2022]. Available: https://www.who.int/data/nutrition/nlis/info/anaemia
11. Zlotkin SH, Davidsson L, Lozoff B. Balancing the Benefits and Risks of Iron Fortification in Resource-Constrained Settings. J Pediatr. 2015;167: S26–S30. doi:10.1016/J.JPEDS.2015.07.017
12. Ngesa O, Mwambi H. Prevalence and Risk Factors of Anaemia among Children Aged between 6 Months and 14 Years in Kenya. PLoS One. 2014;9. doi:10.1371/JOURNAL.PONE.0113756
13. Onyangore FO, Were GM, Mwamburi LA. Prevalence of iron deficiency anaemia and dietary iron intake among infants aged six to nine months in Keiyo South Sub County, Kenya. African Journal of Food, Agriculture, Nutrition and Development. 2016;16. doi:10.18697/ajfand.74.15285
14. Mikulic N, Uyoga MA, Mwasi E, Stoffel NU, Zeder C, Karanja S, et al. The Journal of Nutrition Nutrient Requirements and Optimal Nutrition Iron Absorption is Greater from Apo-Lactoferrin and is Similar Between Holo-Lactoferrin and Ferrous Sulfate: Stable Iron Isotope Studies in Kenyan Infants. doi:10.1093/jn/nxaa226
15. Gruber JF, Gruber LM, Weber RP, Becker-Dreps S, Funk MJ. Rotavirus Vaccine Schedules and Vaccine Response Among Infants in Low- and Middle-Income Countries: A Systematic Review. Open Forum Infect Dis. 2017;4. doi:10.1093/OFID/OFX066
16. Lukowski AF, Koss M, Burden MJ, Jonides J, Nelson CA, Kaciroti N, et al. Iron Deficiency in Infancy and Neurocognitive Functioning at 19 Years: Evidence of Long-Term Deficits in Executive Function and Recognition Memory. Nutr Neurosci. 2010;13: 54. doi:10.1179/147683010X12611460763689
17. World Health Organization. Guideline Daily Iron Supplementation in Infants and Children. : 57.
18. Moraleda C, Rabinovich RN, Menéndez C. Are infants less than 6 months of age a neglected group for anemia prevention in low-income countries? American Journal of Tropical Medicine and Hygiene. 2018. doi:10.4269/ajtmh.17-0487
19. Qian Y, Ying X, Wang P, Lu Z, Hua Y. Early versus delayed umbilical cord clamping on maternal and neonatal outcomes. Archives of Gynecology and Obstetrics. Springer Verlag; 2019. pp. 531–543. doi:10.1007/s00404-019-05215-8
20. Aimone AM, Perumal N, Cole DC. A systematic review of the application and utility of geographical information systems for exploring disease-disease relationships in paediatric global health research: the case of anaemia and malaria. Int J Health Geogr. 2013;12: 1. doi:10.1186/1476-072X-12-1
21. 2019 Kenya Population and Housing Census Reports - CAHF | Centre for Affordable Housing Finance Africa. [cited 10 Oct 2022]. Available: https://housingfinanceafrica.org/documents/2019-kenya-population-and-housing-census-reports/

22. Nyilima;Karanja;Magu;D&Zimmerman. No Title. Journal of African Interdisciplinary Studies(JAIS). 2021;5. Available: https://kenyasocialscienceforum.files.wordpress.com/2021/11/pdf-nyilima-et-al-prevalence-and-maternal-correlates-of-anemia-among-infants-1.pdf
23. Lorenz L, Peter A, Poets CF, Franz AR. E-Mail A Review of Cord Blood Concentrations of Iron Status Parameters to Define Reference Ranges for Preterm Infants. Neonatology. 2013;104: 194–202. doi:10.1159/000353161
24. Kuziga F, Adoke Y, Wanyenze RK. Prevalence and factors associated with anaemia among children aged 6 to 59 months in Namutumba district, Uganda: a cross- sectional study. BMC Pediatr. 2017;17. doi:10.1186/S12887-017-0782-3
25. Domellöf M, Lönnerdal B, Dewey KG, Cohen RJ, Landa Rivera L, Hernell O. Sex differences in iron status during infancy. Pediatrics. 2002;110: 545–552. doi:10.1542/PEDS.110.3.545
26. McClorry S, Zavaleta N, Llanos A, Casapía M, Lönnerdal B, Slupsky CM. Anemia in infancy is associated with alterations in systemic metabolism and microbial structure and function in a sex-specific manner: an observational study. Am J Clin Nutr. 2018;108: 1238–1248. doi:10.1093/AJCN/NQY249
27. Peña-Rosas JP, De-Regil LM, Garcia-Casal MN, Dowswell T. Daily oral iron supplementation during pregnancy. Cochrane Database Syst Rev. 2015;2015: 1–527. doi:10.1002/14651858.CD004736.PUB5
28. Georgieff MK, Krebs NF, Cusick SE. The Benefits and Risks of Iron Supplementation in Pregnancy and Childhood. doi:10.1146/annurev-nutr-082018-124213
29. Moreno-Fernandez J, Ochoa JJ, Latunde-Dada GO, Diaz-Castro J. Iron Deficiency and Iron Homeostasis in Low Birth Weight Preterm Infants: A Systematic Review. Nutrients. 2019;11. doi:10.3390/NU11051090
30. Khan JR, Awan N, Misu F. Determinants of anemia among 6–59 months aged children in Bangladesh: evidence from nationally representative data. BMC Pediatr. 2016;16. doi:10.1186/S12887-015-0536-Z

Table 1: Socio-demographic and health information of the mothers and infants surveyed in Kwale County, Kenya

Factor	Kwale (n=211)	Msambweni (n=213)	Overall (n=424)
Location:			
Mkongani	82 (19.3%)	-	82 (19.3%)
Tsimba Golini	117 (27.6%)	-	117 (27.6%)
Waa	12 (2.8%)	-	12 (2.8%)
Kinondo	-	13 (3.1%)	13 (3.1%)
Ramisi	-	74 (17.5%)	74 (17.5%)
Ukunda	-	100 (23.6%)	100 (23.6%)
Gombato	-	26 (6.1%)	26 (6.1%)
Mother details:			
Age (in years); [mean (min-max)]	27.6 (16-45)	27.8 (18-47)	27.7 (16-47)
Level of education			
None	8 (10.5%)	2 (2.5%)	10 (6.4%)
Primary	41 (54.0%)	44 (54.3%)	85 (54.1%)
Secondary	12 (15.8%)	22 (27.2%)	34 (21.7%)
Post-secondary	15 (19.7%)	13 (16.1%)	28 (17.8%)
Parity; [median (min-max)]	3 (1-11)	3 (1-8)	3 (1-11)
Haemoglobin (g/dl); [mean (min-max)	10.8(7.7-15.0)	10.8 (7.0-14.0)	10.8 (7.0-15.0)
Weight (in kg); [mean (min-max)	58.2 (39.4-95.2)	61.5 (35-112)	59.5 (35-112)
Use iron supplement			
Yes	75 (98.7%)	79 (97.5%)	154 (98.1%)
No	1 (1.3%)	2 (2.5%)	3 (2%)
Number of ANC visits; [mean (min-max)]	4 (2-8)	5 (2-9)	5 (2-9)
Had twin pregnancy			
Yes	1 (1.3%)	2 (2.5%)	3 (2.0%)
No	75 (98.7%)	79 (97.5%)	154 (98.1%)
Household water source			
Improved	50 (65.8%)	59 (72.3%)	109 (69.4%)
Unimproved	26 (34.2%)	22 (27.2%)	48 (30.6%)
Cost of travel to the nearest health facility (Ksh); [median (min-max)]	126 (50-300)	117 (50-500)	122 (50-500)
Infant details:			
Gender			
Male	108 (51.4%)	124 (58.5%)	232 (55%)
Female	102 (48.6%)	88 (41.5%)	190 (45%)
Weight at birth (in kg); [mean (min-max)]	3.1 (2.1-5.2)	3.1 (2.0-4.9)	3.10 (2-5.2)
Weight at 6 weeks (in kg); [mean (min-max)]	4.6 (3.5-6.1)	4.7 (3.4-6.5)	4.7 (3.4-6.5)
Gestation (in weeks); [mean (min-max)]	37.9 (34-40)	37.7 (32-42)	37.8 (32-42)
Height (in cm); [mean (min-max)]	54.7 (48-60.3)	54.7(48.5-61.4)	54.7 (48-61.4)
Head circumference (in cm); [mean (min-max)]	38.1 (35-49)	37.8(35-54.4)	37.9 (35-54.4)

Wasting			
Not wasted	204 (96.7%)	207 (99%)	411 (97.9%)
Wasted	7 (3.3%)	2 (1%)	9 (2.1%)
Underweight			
Not underweight	210 (99.5%)	209 (100%)	419 (99.8%)
Underweight	1 (0.5%)	0	1 (0.2%)
Stunting			
Not stunted	202 (95.7%)	192 (91.9%)	394 (93.8%)
Stunted	9 (4.3%)	17 (8.1%)	26 (6.2%)
Haemoglobin; [mean (min-max)	125.6 (95-162)	118.6 (87-170)	122.1 (87-170)
Zinc protoprophyrin; [mean (min-max)	42.6 (19-125)	45.4 (16-90)	44 (16-125)

Table 2: Proportion of infants with selected nutritional conditions

Nutritional conditions	Wasting % (95%CI)	Underweight % (95%CI)	Stunting % (95%CI)	Iron deficient % (95%CI)	Anaemic % (95%CI)	Iron deficiency Anemia % (95%CI)
Overall	2.1% (1.1-4.1); n=9	0.2% (0.0-1.7); n=1	6.2% (4.3-9.0); n=26	60.4% (55.9-65.2); n=256	21.0% (17.5-25.2); n=89	15.8% (12.7-19.7); n=67
SubCounty						
Kwale	3.3% (1.6-6.9); n=7	0.5% (0.1-3.4); n=1	4.3% (2.3-8.1); n=9	55.1% (49.1-62.6); n=117	12.8% (9.0-18.2); n=27	9.5% (6.2-14.4); n=20
Msambweni	1.0% (0.2-3.8); n=2	0%	8.1% (5.2-12.8); n=17	65.3% (59.2-72.0); n=139	29.1% (23.6-35.9); n=62	22.1% (17.1-28.4); n=47
Location:						
Mkongani	2.4% (0.6-9.6); n=2	0%	4.9% (1.9-12.7); n=4	59.6% (50.0-71.4); n=49	14.6% (8.7-24.7); n=12	9.8% (5.1-18.8); n=8
Tsimba Golini	4.3% (1.8-10.1); n=5	0.9% (0.1-6.0); n=1	4.3% (1.8-10.1); n=5	50.4% (42.1-60.4); n=59	12.8% (8.0-20.6); n=15	10.3% (6.0-17.5); n=12
Waa	0%	0%	0%	75.0% (51.1-104); n=9	0%	0%
Kinondo	0%	0%	0%	61.5% (40.0-94.6); n=8	50.8% (13.6-69.5); n=4	15.4% (4.3-55); n=2
Ramisi	2.7% (0.7-10.7); n=2	0%	11.0% (5.7-21.1); n=8	66.2% (56.3-78); n=49	27.0% (18.6-39.3)	24.3% (16.3-36.4); n=18
Ukunda	0%	0%	7.1% (3.5-14.6); n=7	62.0% (53.2-72.3); n=62	27.0% (19.6-37.3); n=27	18.0% (11.8-27.3); n=18
Gombato	0%	0%	7.7% (2.0-29.1); n=2	77.0% (62.3-94.9); n=20	42.3% (27.0-66.3); n=11	34.6% (20.4-58.7); n=9
Age of the mother						
≤20	0%	2.1% (0.3-14.8); n=1	6.4% (2.1-19.1); n=3	72.3% (60.6-86.3); n=34	19.1% (10.6-34.5); n=9	17.0% (9.1-32.0); n=8
21-30	2.1% (0.9-4.9); n=5	0%	5.8% (3.5-9.6); n=14	61.9% (56.1-68.3); n=151	20.5% (16.0-26.2); n=50	14.8% (10.9-19.-9); n=36
31-40	3.4% (1.3-9.0); n=4	0%	6.0% (2.9-12.3); n=7	52.1% (43.9-61.9); n=62	24.4% (17.8-33.4); n=29	18.5% (12.7-27.0); n=22
>40	0%	0%	16.7% (4.7-59.1); n=2	75.0% (54.104); n=9	8.3% (1.3-54.4); n=1	8.3% (1.3-54.4); n=1
Mother's level of education						
None	0%	0%	0%	60.0% (36.2-99.5); n=6	10.0% (1.6-64.2); n=1	10.0% (1.6-64.2); n=1
Primary	2.4% (0.6-9.3); n=2	0%	3.5% (1.2-10.7); n=3	62.4% (52.9-73.6); n=53	22.4% (15.0-33.2); n=19	14.1% (8.4-23.8); n=12
Secondary	0%	0%	0%	50% (35.7-70); n=17	11.8% (4.7-29.5); n=4	8.8% (3.0-26.0); n=3
Post-secondary	11.5% (4.0-33.4); n=3	0%	15.5% (6.2-37.9); n=4	64.3% (48.8-84.7); n=18	21.4% (10.5-43.6); n=6	17.9% (8.1-39.5); n=5
Parity						

1	2.2% (0.6-8.7); n=2	1.1% (0.2-7.7); n=1	8.8% (4.5-17); n=8	61.3% (52.1-72.0); n=57	23.7% (16.4-34.1); n=22	18.3% (11.9-28.1); n=17
2	2.7% (0.9-8.2); n=3	0%	3.6% (1.4-9.3); n=4	60.7% (52.3-70.5); n=68	18.8% (12.8-27.6); n=21	12.5% (7.7-20.4); n=14
3	1.1% (0.2-7.9); n=1	0%	5.6% (2.4-13.2); n=5	62.9% (53.6-73.8); n=56	21.3% (14.3-31.8); n=19	19.1% (12.5-29.3); n=17
≥4	2.4% (0.8-7.4); n=3	0%	5.6% (2.7-11.6); n=7	60.0% (52.-69.2); n=75	21.6% (15.5-30.2); n=27	15.2% (10.0-23.0); n=19
Mother anemic						
Yes	2.9% (1.2-7.0); n=5	0.6% (0.1-4.2%); n=1	6.5% (3.7-11.5); n=11	63.0% (56.2-70.6); n=109	22.0% (16.6-29.1); n=38	17.9% (13.0-24.7); n=31
No	1.2% (0.3-4.9); n=2	0%	6.8% (3.9-12.1); n=11	67.1% (60.2-74.7); n=108	21.7% (16.2-29.1); n=35	16.2% (11.4-23.0); n=26
Weight of the mother						
<50	1.0% (0.1-7.2); n=1	0%	5.1% (2.2-12.0); n=5	61.2% (52.3-71.7); n=60	17.3% (11.3-26.7); n=17	14.3% (8.8-23.2); n=14
50-59	0.7% (0.1-4.9); n=1	0%	4.9% (2.4-10.1); n=7	58.2% (50.7-66.8); n=85	26% (19.8-34.2); n=38	19.2% (13.8-26.8); n=28
60-69	3.1% (1.0-9.3); n=3	1% (0.1-7.2); n=1	8.2% (4.2-15.9); n=8	56.1% (47.1-66.9); n=55	20.4% (13.8-30.2); n=20	16.3% (10.4-25.6); n=16
70-79	4.3% (1.1-16.5); n=2	0%	6.4% (2.1-19.1); n=3	68.1% (56.0-82.8); n=32	25.5% (15.7-41.6); n=12	14.9% (7.5-29.5); n=7
>80	5.9% (1.5-22.6); n=2	0%	8.8% (3.0-26); n=3	70.6% (56.8-87.8); n=24	5.9% (1.5-22.6); n=2	5.9% (1.5-22.6); n=2
Mother uses iron supplement						
Yes	3.3% (1.3-7.8); n=5	0%	4.6 % (2.2-9.5); n=7	60.4% (53.1-68.6); n=93	18.2% (13.0-25.4); n=28	13.6% (9.2-20.3); n=21
No	0%	0%	0%	33.3% (6.7-165.1); n=1	66.7% (30.0-148.4); n=2	0%
Infant gender						
Male	3.9% (2.1-7.5); n=9	0.4% (0.1-3.1); n=1	5.7% (3.3-9.6); n=13	64.2% (58.3-70.7); n=149	26.7% (21.6-33.1); n=62	20.3% (15.7-26.2); n=47
Female	0%	0%	6.8% (4.0-11.6); n=13	56.3% (49.7-63.8); n=107	14.2% (10.0-20.2); n=27	10.5% (7.0-15.9); n=20
Infant weight at birth						
<2.5	3.2% (0.5-22.2); n=1	0%	32.3% (19.4-53.7); n=10	71% (56.7-88.9); n=22	29% (16.7-50.3); n=9	22.3% (11.8-43.3); n=7
≥2.5	2.1% (1.0-4.1); n=8	0.3% (0.0-1.8); n=1	4.2% (2.6-6.7); n=16	59.4% (54.7-64.5); n=230	20.2% (16.5-24.6); n=78	15% (11.8-19.0); n=58
Infant weight at 6 weeks						
<5.0	3.0% (1.6-5.8); n=9	0.3% (0.0-2.4); n=1	8.8% (6.1-12.7); n=26	59.3% (53.9-65.1); n=176	19.9% (15.8-25); n=59	16.2% (12.5-20.9); n=48
≥5.0	0%	0%	0%	63.5% (55.6-72.5); n=80	23.8% (17.4-32.5); n=30	15.1% (1-22.8); n=19
Gestational weeks						
<37	2.0% (0.3-13.6); n=1	0%	2.0% (0.3-13.7); n=1	60.4% (48.5-75.1); n=32	24.5% (15.3-39.3); n=13	18.9% (10.8-33.0); n=10
≥37	2.2% (1.1-4.3); n=8	0.3% (0.0-1.9); n=1	6.6% (4.5-9.7); n=24	60.9% (56.1-66.1); n=223	20.5% (16.7-25.1); n=75	15.6% (12.3-19.8); n=57
Infant's height (cm)						
<55	0.5% (0.1-3.5); n=1	0.5% (0.1-3.5); n=1	13.1% (9.1-18.7); n=26	59.5% (53.1-66.7); n=119	22.0% (16.9-28.6); n=44	17.0% (12.5-23.1); n=34
≥55	3.6% (1.8-7.1); n=8	0%	0%	61.4% (55.4-68.2); n=137	20.2% (15.5-26.2); n=45	14.8% (10.8-20.3); n=33
Infant's head circumference						
<38	2.2% (1.0-4.9); n=6	0.4% (0.1-2.6); n=1	9.3% (6.4-13.5); n=25	63.5% (58.0-69.5); n=172	20.3% (16.0-25.7); n=55	15.9% (12.1-20.9); n=43
≥ 38	2.0% (0.6-6.1); n=3	0%	0.7% (0.1-4.7); n=1	55.3% (47.9-63.8); n=84	22.4% (16.6-30.1); n=34	15.8% (10.9-22.8); n=24

Table 3: Chi-square association of anaemia with selected predictors among six-week-old infants in Kwale County, Kenya.

Selected predictors	Iron deficient		Anaemic		Iron deficiency anemia	
	Yes	No	Yes	No	Yes	No
SubCounty						
Kwale	117	94	27	184	20	191
Msambweni	139	74	62	151	47	166
χ^2(test statistic, p-value)	χ^2 =4.26, df=1, p=0.039*		χ^2 =17.00, df=1, p=0.000*		χ^2 =12.62, df=1, p=0.000*	
Age of the mother						
≤20	34	13	9	38	8	39
21-30	151	93	50	194	36	208
31-40	62	57	29	90	22	97
>40	9	3	1	11	1	11
χ^2(test statistic, p-value)	χ^2 =7.53, df=3, p=0.057		χ^2 =2.10, df=3, p=0.552		χ^2 =1.40, df=3, p=0.707	
Mother's level of education						
None	6	4	1	9	1	9
Primary	53	32	19	66	12	73
Secondary	17	17	4	30	3	31
Post-secondary	18	10	6	22	5	23
χ^2(test statistic, p-value)	χ^2 =1.82, df=3, p=0.610		χ^2 =2.40, df=3, p=0.494		χ^2 =1.23, df=3, p=0.745	
Parity						
1	57	36	22	71	17	76
2	68	44	21	91	14	98
3	56	33	19	70	17	72
≥4	75	40	27	98	19	106
χ^2(test statistic, p-value)	χ^2 =0.20, df=3, p=0.978		χ^2 = 0.75, df=3, p=0.861		χ^2 = 2.08, df=3, p=0.556	
Mother anemic						
Yes	109	64	38	135	31	142
No	108	53	35	126	26	135
χ^2(test statistic, p-value)	χ^2 =0.61, df=1, p=0.435		χ^2 =0.0025, df=1, p=0.960		χ^2 =0.18, df=1, p=0.667	
Mother uses iron supplement						
Yes	93	61	28	126	21	133
No	1	2	2	1	0	3
χ^2(test statistic, p-value)	χ^2 =0.90, df=1, p=0.344		χ^2 =4.48, df=1, p=0.034*		χ^2 =0.47, df=1, p=0.492	
Infant gender						
Male	149	83	62	170	47	185
Female	107	83	27	163	20	170
χ^2(test statistic, p-value)	χ^2 =2.74, df=1, p=0.098		χ^2 =9.83, df=1, p=0.002*		χ^2 =7.41, df=1, p=0.006*	
Infant weight at birth						
<2.5	22	9	9	22	7	24
≥2.5	230	157	78	309	58	329

χ^2(test statistic, p-value)	χ^2 =1.60, df=1, p=0.207		χ^2 =1.37, df=1, p=0.241		χ^2 =1.26, df=1, p=0.262	
Gestational weeks						
<37	32	21	13	40	10	43
≥37	223	143	75	291	57	309
χ^2(test statistic, p-value)	χ^2 =0.01, df=1, p=0.939		χ^2 =0.45, df=1, p=0.500		χ^2 =0.37, df=1, p=0.541	
Iron deficient						
Yes			67	189	67	189
No			22	146	0	168
χ^2(test statistic, p-value)			χ^2 =10.46, df=1, p=0.001*		5χ^2 =52.22, df=1, p=0.000*	

Table 4: Univariable logistic regression analysis of factors associated with anemia among six-week-old infants in Kwale County, Kenya.

**Factors**	**N (%)**	**Odds Ratio (95% CI)**	**P-value**
Infant's factors:			
Gestation weeks			
<37 weeks		1.26 (0.64-2.48)	0.501
≥37 weeks		Reference	
Gender			
Male		2.20 (1.33-3.63)	0.002*
Female		Reference	
Birth weight			
<2.5kg		1.62 (0.72-3.66)	0.245
≥ 2.5kg		Reference	
Wasted			
< 2.0 SD	9 (2.1%)	Insuff obs	-
≥ 2.0 SD	411 (97.9%)	Reference	
Underweight			
<2.0 SD	1 (0.2%)	Insuff obs	-
≥ 2.0SD	419 (99.8%)	Reference	
Stunted			
< 2.0 SD	26 (6.2%)	1.16 (0.45-2.98)	0.759
≥ 2.0 SD	394 (93.8%)	Reference	
Weight at 6 weeks			
<5.0kg		0.79 (0.48-1.31)	0.363
≥ 5.0kg		Reference	
Height at 6 weeks			
<55		1.12 (0.70-1.78)	0.647
≥55		Reference	
Head circumference			

<38		0.88 (0.56-1.43)	0.616
≥38		Reference	
Iron deficient			
Yes		2.35 (1.39-3.99)	0.001*
No		Reference	
Maternal factors:			
Anemic			
Yes		1.01 (0.60-1.70)	0.960
No		Reference	
Use of iron supplements			
Yes		Reference	
No		9.0 (0.79-102.8)	0.077
Age			
≤20		Reference	
21-30		1.09 (0.49-2.40)	0.834
31-40		1.36 (0.59-3.15)	0.472
>40		0.38 (0.04-3.37)	0.388
Weight			
<50		Reference	
50-59		1.68 (0.88-3.18)	0.114
60-69		1.22 (0.60-2.50)	0.584
70-79		1.63 (0.71-3.78)	0.251
≥80		0.30 (0.07-1.36)	0.119
Parity			
1		Reference	
2		0.74 (0.4-1.5)	0.391
3		0.88 (0.4-1.8)	0.710
≥4		0.89 (0.47-1.69)	0.719
Education			
None		Reference	
Primary		2.59 (0.31-25.8)	0.381
Secondary		1.19 (0.12-12.14)	0.877
Post-secondary		2.45 (0.26-25.39)	0.435
SubCounty:			
Kwale		Reference	
Msambweni		2.80 (1.70-4.62)	<0.001*
*Indicates statistically significant factors (p<0.05)			

Table 5: Pairwise correlation of factors associated with anemia among six-week-old infants in Kwale County, Kenya

	ANM	GES	PAR	BW	GEN	IDA	WAS	UWT	STU	AMO	UIS	AGM	WOM	EDS	WOC	HOC	HCC
ANM	1.0000																
GES	0.0329	1.0000															
PAR	-0.0329	-0.0571	1.0000														
BW	0.0573	-0.0234	0.0068	1.0000													
GEN	-0.1526	0.0602	-0.0360	0.1107	1.0000												
IDA	0.1571	-0.0038	-0.0145	0.0618	-0.0805	1.0000											
WAS	-0.0756	-0.0052	0.0114	0.0206	-0.1350	-0.0493	1.0000										
UWT	0.0956	-0.0183	-0.0624	-0.0140	-0.0446	0.0393	-0.0072	1.0000									
STU	0.0150	-0.0637	-0.0276	0.3048	0.0240	0.0448	0.0302	0.1902	1.0000								
AMO	0.0027	-0.0582	0.0449	0.0552	0.0148	-0.0427	0.0590	0.0536	-0.0073	1.0000							
UIS	0.1688	-0.0571	0.1548	-0.0404	-0.1347	-0.0756	-0.0256	.	-0.0306	-0.1686	1.0000						
AGM	0.0151	-0.0536	0.6186	-0.0289	0.0064	-0.0880	0.0483	-0.0887	0.0318	0.0245	0.0054	1.0000					
WOM	-0.0401	-0.0040	0.0803	-0.1243	0.0573	0.0503	0.1074	0.0218	0.0486	-0.1651	-0.0279	0.1856	1.0000				
EDS	-0.0054	0.1213	-0.3322	0.0237	0.0377	-0.0136	0.1533	.	0.1679	-0.1685	-0.0287	0.0161	0.2331	1.0000			
WOC	-0.0443	0.0460	0.0334	0.1427	0.1741	-0.0396	0.0958	0.0316	0.1663	0.0824	-0.1319	-0.0486	-0.1808	-0.0788	1.0000		
HOC	-0.0223	-0.0318	-0.0289	-0.2055	-0.1808	0.0198	0.1075	-0.0515	-0.2707	-0.0393	-0.0583	0.0511	0.1698	0.0699	-0.4408	1.0000	
HCC	0.0244	-0.0930	-0.0441	-0.1344	-0.1532	-0.0805	-0.0081	-0.0366	-0.1719	-0.0333	0.0124	0.0414	0.0810	-0.0398	-0.2771	0.3244	1.0000

ANM: Anemia, GES: Gestational weeks, PAR: Parity, BW: Birth weight, GEN: Gender, IDA: Iron deficient anemia, WAS: Wasting, UWT: Underweight, STU: Stunted, AMO: Anemic mother, UIS: Use of iron supplements, AGM: Age of the mother, WOM: Weight of the mother, EDS: Education level, WOC: Weight of the child, HOC: Height of the child, HCC: Head circumference of the child.

Table 6: Multivariable logistic regression analysis of factors associated with anemia among six-week-old infants in Kwale County, Kenya

Factors	Adjusted Odds Ratio (95% CI)	P-Value
Infant’s factors:		
Gestation weeks		
<37 weeks	0.65 (0.15-2.82)	0.562
≥37 weeks	Reference	
Gender		
Male	1.92 (0.66-5.54)	0.229
Female	Reference	
Birth weight		
<2.5kg	1.28 (0.20-8.25)	0.798
≥ 2.5kg	Reference	
Stunted		
< 2.0 SD	0.20 (0.01-3.86)	0.285

≥ 2.0 SD	Reference	
Weight at 6 weeks		
<5.0kg	1.14 (0.31-4.19)	0.844
≥ 5.0kg	Reference	
Height at 6 weeks		
<55	1.31 (0.41-4.22)	0.652
≥55	Reference	
Head circumference		
<38	0.76 (0.25-2.36)	0.639
≥ 38	Reference	
Iron deficient		
Yes	2.15(0.68-6.81)	0.192
No	Reference	
Maternal factors:		
Use of iron supplements		
Yes	Reference	
No	74.01(2.45-2238.21)	0.013*
Age		
≤20	Reference	
21-30	1.65(0.19-14.36)	0.649
31-40	11.37(0.69-186.86)	0.089
>40		
Weight		
<50	Reference	
50-59	1.80(0.39-8.43)	0.454
60-69	1.81(0.37-8.88)	0.463
70-79	1.05(0.16-6.93)	0.963
≥80	0.24(0.01-4.14)	0.330
Parity		
1	Reference	
2	0.52(0.09-3.03)	0.464
3	0.38(0.05-2.57)	0.318
≥4	0.06(0.00-0.69)	0.024*
Education		
None	Reference	
Primary	3.10(0.28-34.02)	0.354
Secondary	0.53(0.04-7.88)	0.646
Post-secondary	2.82(0.17-47.02)	0.470
Subcounty:		
Kwale	Reference	
Msambweni	2.28(0.78-6.62)	0.129

*Indicates statistically significant factors (p<0.005)

List of figures

Figure 1: Prevalence of ID, anemia and IDA among six-week-old infants by locations in Kwale County, Kenya.

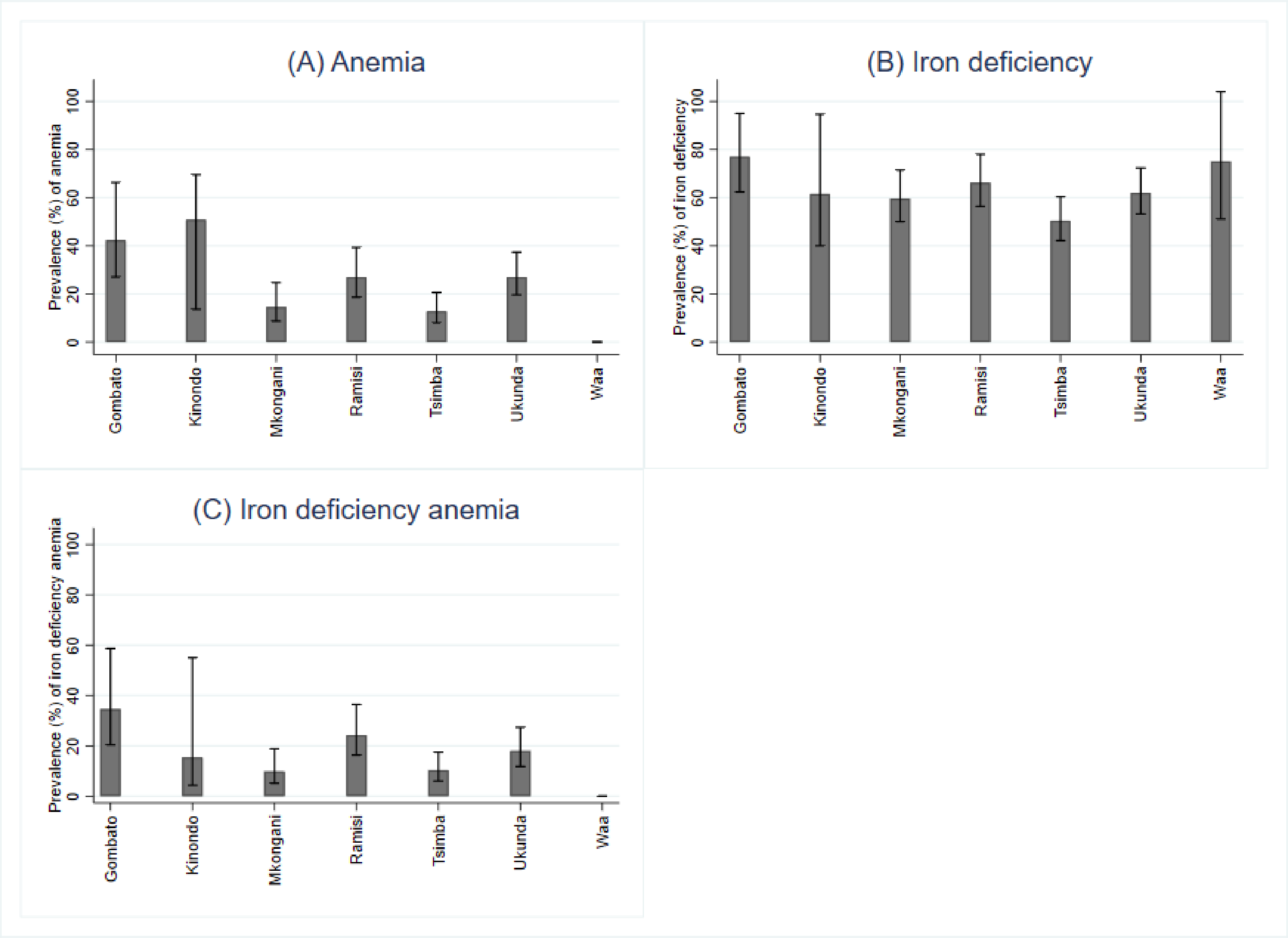

Supplement: Attachment — Submitted filename: PGPH-D-23-01767_reviewer.pdf [file pgph.0003062.s002.pdf]
