# Supplementary material for: Prevalence and predictors of anemia among six-week-old infants in Kwale County, Kenya: A cross-sectional study
Source: PLOS Glob Public Health. 2024 Mar 29;4(3):e0003062. doi: 10.1371/journal.pgph.0003062 (PMC10980238; doi:10.1371/journal.pgph.0003062)
Supplement: Attachment — Submitted filename: Response to reviewers.docx [file pgph.0003062.s003.docx]

Response to reviewers:

Reviewer #1: Firstly, I would congratulate the authors for studying an important issue of public health relevance. There are very few treatment regimens and recommendations in the under 6 months age group, hence the authors have tried throwing light on the gaps in this area. The findings will be very valuable for policy and action.
The authors have done good job of extracting maximum information for this nested study from the parent study. However I feel that the paper would benefit from minor editing.

>> Thank you finding value in our work.

Minor edits need to be done as follows:

General comments: The references in the text need to mentioned by their number rather than the authors. This uniformity needs to be maintained throughout the manuscript.

>> We agree - This has been done.

All the significant values in the tale need to be highlighted for easier reading.

>> We agree - All significant values in the tables have been bolded. See Tables
3, 4 and 6.

Specific comments:

Introduction:
Line number 71, 72 the reference number needs to be mentioned rather than the author names, similarly at line number 101, 102, 125, 126

>> We agree - All citation numbers have been added.

Materials and methods:
Line 137 reference of the RCT needs to be mentioned

>> We agree - This has been added.

Results:
It has been mentioned in the methods as well as the results that the Hemoglobin at the mother’s last ANC visit was recorded and that value is used in all the analysis. I would add more value if you could mention the average weeks at this visit or at what weeks this last ANC visit was.

>> We agree that this information would have been valuable, but unfortunately, the date and average gestation weeks of the last ANC visit was not collected.

This study is a nested study and there was no maternal blood sample but certainly if instead of just recorded values of Hemoglobin if there was a maternal blood sample available it would have added value to this study

>> We agree, but maternal blood sample was not collected.

Line 281, 282 you have mentioned that mothers weighing 50-59 kg had higher proportion of anaemic infants than mothers weighing >80kg so does that mean that the in between range of weight the relation was not significant? Need to clarify

>> We have rewritten this statement to show that we are not comparing the proportions within the categories. See lines 263-265.

Line 288,289 There is a difference between the 2 counties’, is there any specific reason which can be identified, is the diet different in these 2 counties? There is no direct measure of diet in this study which also is one of the drawbacks as diet plays a important role in anaemia. Some measure of diet or some general information collected through and Focus Group Discussion would have given clarity on whether there were any major differences in the diet.

>> Although we did not collect dietary information that would have helped improve the presentation of the results, we have thoroughly discussed the importance of maternal diet influencing infants’ health outcomes including anemia. We have also mentioned this as a general limitation of the study. See lines 341-357.

Reviewer #2: The manuscript was decently written. However, there are places that can be improved before moving forward -
1) For statistical analysis, there are confidence intervals with very wide bounds which usually indicate problems with the models. The authors need to scrutinize them in revision.

>> Thank you, we have rechecked our models and feel they are sound. The wider confidence intervals in the paper for some variables reflects the small number of observations for that variable. For example, on use of iron supplements during pregnancy, only 157 mothers gave this information and, only 3 mothers reported that they did not use iron supplements during pregnancy.

2) For data availability, it seems the raw dataset used for the analysis has not been uploaded. To improve reproducibility, the authors can consider providing it.

>> We indicated that data will be made available upon request. See line 419. However, because the data used to complete this analysis are the baseline data of the main intervention study which is still ongoing, we prefer not to upload the raw dataset at the moment. We may do this when the study is completed and all data analyzed.

Other comments, along with their corresponding sections highlighted, are in the uploaded attachment.

>> We agree with all the reviewers’ suggestions and edits that were in the uploaded attachment and our responses to these are shown in the revised manuscript as track changes.
